# Identifying the spatial pattern and driving factors of nitrate in groundwater using a novel framework of interpretable stacking ensemble learning

**DOI:** 10.1007/s10653-024-02201-1

**Published:** 2024-10-29

**Authors:** Xuan Li, Guohua Liang, Lei Wang, Yuesuo Yang, Yuanyin Li, Zhongguo Li, Bin He, Guoli Wang

**Affiliations:** 1 School of Hydraulic Engineering, Dalian University of Technology, Dalian, 116024 China; 2 British Geological Survey, Keyworth, Nottingham, NG12 5GG UK; 3 Key Laboratory of Groundwater Resources and Environment, Ministry of Education, Jilin University, Changchun, 130021 China; 4 Liaoning Water Affairs Service Center, Shenyang, 110003 China; 5 Department of Geography, Durham University, Durham, DH1 3LE UK

**Keywords:** Water quality, Groundwater, Spatial distribution, Driving factors, Ensemble learning, Interpretable machine learning

## Abstract

Groundwater nitrate contamination poses a potential threat to human health and environmental safety globally. This study proposes an interpretable stacking ensemble learning (SEL) framework for enhancing and interpreting groundwater nitrate spatial predictions by integrating the two-level heterogeneous SEL model and SHapley Additive exPlanations (SHAP). In the SEL model, five commonly used machine learning models were utilized as base models (gradient boosting decision tree, extreme gradient boosting, random forest, extremely randomized trees, and k-nearest neighbor), whose outputs were taken as input data for the meta-model. When applied to the agricultural intensive area, the Eden Valley in the UK, the SEL model outperformed the individual models in predictive performance and generalization ability. It reveals a mean groundwater nitrate level of 2.22 mg/L-N, with 2.46% of sandstone aquifers exceeding the drinking standard of 11.3 mg/L-N. Alarmingly, 8.74% of areas with high groundwater nitrate remain outside the designated nitrate vulnerable zones. Moreover, SHAP identified that transmissivity, baseflow index, hydraulic conductivity, the percentage of arable land, and the C:N ratio in the soil were the top five key driving factors of groundwater nitrate. With nitrate threatening groundwater globally, this study presents a high-accuracy, interpretable, and flexible modeling framework that enhances our understanding of the mechanisms behind groundwater nitrate contamination. It implies that the interpretable SEL framework has great promise for providing valuable evidence for environmental management, water resource protection, and sustainable development, particularly in the data-scarce area.

**Supplementary Information:**

The online version contains supplementary material available at 10.1007/s10653-024-02201-1.

## Introduction

Groundwater is a valuable resource, serving as the primary source of drinking water for over a third of the population in the world (IAHS, 2023). However, with the increasing human activities, excess nitrogen released into the subsurface environment causes groundwater nitrate contamination (Castaldo et al., 2021; Liu et al., 2023; Mahlknecht et al., 2023). It poses a threat to human health and environmental security, which has attracted global attention (Kaur et al., 2020; Knoll et al., 2019; Ransom et al., 2022). Nitrate ingestion by humans is related to methemoglobinemia, adverse pregnancy outcomes, thyroid disease, and specific cancers (Picetti et al., 2022; Richards et al., 2022). Due to the importance of protecting public health, the World Health Organization (WHO) set the guideline value of 50 mg/L NO_3_ (equivalent to 11.3 mg/L-N) for nitrate concentration in drinking water (WHO, 2022). Therefore, it is crucial to protect groundwater from nitrate pollution and limit nitrogen inputs. To achieve the goal, it is necessary to identify the spatial pattern and important influential factors of groundwater nitrate.

The Eden Valley is a largely rural area in the UK, and groundwater is widely used for public water supply, industry, and minor private supplies for farms (Butcher et al., 2003). Nevertheless, groundwater nitrate pollution is a serious problem in the study area, which is primarily caused by intensive farming practices (Wang & Burke, 2017). The extensive application of fertilizers and manure in arable land in the 1980s significantly increased nitrogen levels in the soil (Wang et al., 2012). Moreover, it is reported that atmospheric nitrogen deposition is recognized as an important nitrogen source for woodland soils in the UK (Vanguelova et al., 2024). Nitrogen can be converted into nitrate through nitrification and then leach into aquifers via infiltration, posing a severe threat to groundwater quality. Notably, in areas with a thick unsaturated zone in the Eden Valley, the peak nitrogen loading has not reached the groundwater table (Wang et al., 2013). To protect waters against nitrate pollution, the EU proposed Nitrates Directive 91/676/EEC in 1991, which requires the designation of certain areas as Nitrate Vulnerable Zones (NVZs) where nitrate in surface water or groundwater has exceeded or could exceed 50 mg/L nitrate (11.3 mg/L-N) due to agricultural sources, and deliver measures (EU, 1991; Musacchio et al., 2020). The recent Nitrate Vulnerable Zones (NVZs) designation in 2021 delineated four groundwater NVZs in the Eden Valley (EA, 2021). To address the groundwater nitrate pollution in the study area, it is crucial to investigate the spatial distribution of groundwater nitrate concentrations and gain a thorough understanding of the impacts of environmental variables.

Accurate groundwater quality spatial distribution is essential for comprehending current contaminant levels, particularly for the data-scarce area. However, conventional spatial interpolation methods typically depend on geographical information while neglecting the impacts of environmental factors (Mainali et al., 2019), which can result in potential high deviation and uncertainty in predictions. On the other hand, frequent water quality monitoring and testing is costly and time-consuming, and data availability is often delayed (Li et al., 2022). By contrast, machine learning (ML) is a new data-driven model that can identify the complex and non-linear relationship between input and target variables, which has developed rapidly in recent decades. With the advantages of high accuracy, low cost, and time-saving, ML has been increasingly applied in groundwater investigations and has shown promising results (Barzegar et al., 2021; Iqbal et al., 2023; Nadiri et al., 2023; Ransom et al., 2022).

Nevertheless, it is inevitable that individual ML models may selectively capture local patterns and be prone to noise or errors, which can lead to poor performance on unseen data. In addition, although ML has shown promise in predicting variables, its complex structure, like an intelligent black-box, presents challenges in understanding the mechanisms (Nearing et al., 2021), such as support vector regression (SVR) with a non-linear kernel and artificial neural network (ANN) with multiple hidden layers, in particular for the ensemble learning model within a multi-layer structure. Otherwise, ranking the features through multiple transformations is essentially meaningless. Tree-based models, like extreme gradient boosting (XGB) and random forest (RF), enable interpretability of the model; whereas, their explanations are limited to the training data, and XGB can only offer the global explanation. This hinders water managers from leveraging machine learning predictions to formulate targeted safeguard policies.

To tackle the dual challenge of predictive performance and interpretability, combining stacking and the interpretable method offers a potential solution. Stacking ensemble learning (SEL) is a powerful ensemble learning method, and it can enhance overall prediction accuracy by integrating the outputs of multiple base models to obtain the final prediction based on the “wisdom of crowds” (Wang et al., 2021). To decrease the risk of overfitting, it is commonly coupled with cross-validation (CV) to generate new training data for the meta-model. The SEL model exhibits great promise of applications in many fields, e.g., hydrology (Lu et al., 2023; Shams et al., 2021), meteorology (Gu et al., 2022; Morshed-Bozorgdel et al., 2022), and environment (Sakizadeh et al., 2024; Wang et al., 2021). Given its superior model performance and generalization in previous studies, the SEL model is required to be introduced to accurately predict groundwater contamination, especially in the data-scarce area. On the other hand, Shapely addictive explanations (SHAP) is an advanced interpretable method that can not only provide global explanations and feature importance but also explain an individual prediction (Lundberg et al.; Lundberg & Lee, 2017). It can also identify the positive and negative effects on predictive results, as well as linear and nonlinear relationships. Thus, SHAP is a valuable tool in enhancing model transparency and interpretability, facilitating a deeper insight into the ML model (Li et al., 2022). However, it is rarely used in groundwater pollution research.

In this study, we adopt a two-level heterogeneous SEL model, consisting of five base models at level 0 (gradient boosting decision tree (GBDT), XGB, RF, extremely randomized trees (ET), and k-nearest neighbor (KNN)), and a meta-model at level 1 (KNN) that uses the output from the base models. SHAP is employed to identify important driving factors and quantify their contributions. To our knowledge, the SEL model combined with the interpretable ML method has not been used to analyze contaminants in water before, and this study attempts to fill this gap.

The main objectives of this study are to (1) develop a novel two-level interpretable stacking ensemble learning (ISEL) framework for analyzing groundwater nitrate; (2) compare the model performance and generalization ability of the SEL model to five individual ML models; (3) map the spatial distribution of nitrate in groundwater and pinpoint high nitrate areas in the Eden Valley, UK; and (4) identify key driving factors of groundwater nitrate and quantitatively analyze their influence.

## Data and method

### Study area

**Fig. 1:**
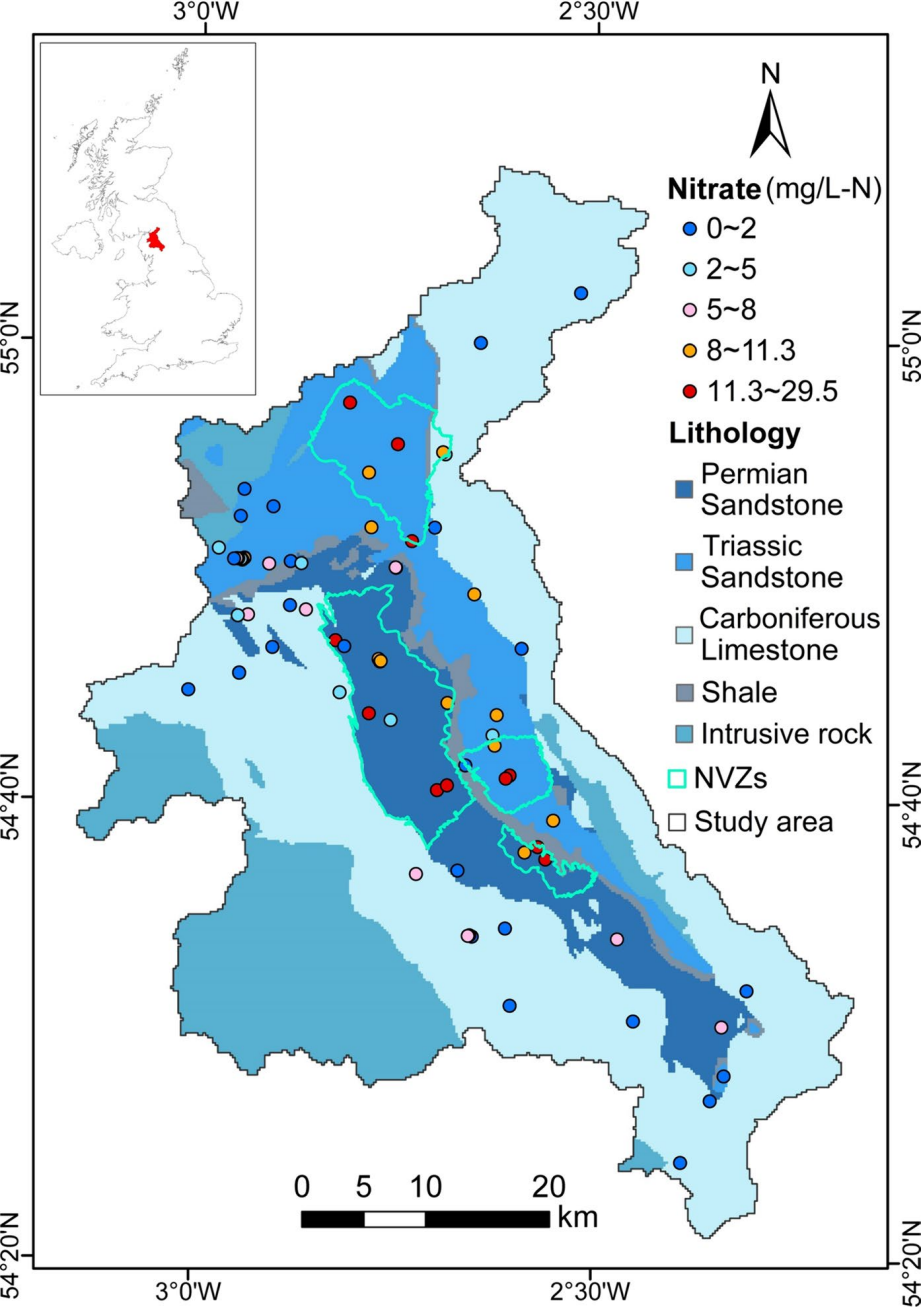
Lithology, well locations, groundwater nitrate concentrations, and NVZs in the study area

The Eden Valley is located in Cumbria, North West England, covering approximately 2308 km^2^ (Fig. 1). The River Eden origins from the Pennines and discharges into the Solway Firth in the northwest, running northwards and joined by tributary rivers, such as the River Eamont, the River Irthing, and the River Caldew. The meteorological, hydrology, and hydrogeology conditions in the Eden Valley are shown in Fig. S1. In the study area, the elevation varies from 945 m to the sea level, which is relatively high in the southwest and the east but low in the valley. It has a temperate marine climate, with an average annual precipitation of approximately 1000 mm/a in the study area and exceeding 1500 mm/a on higher ground (Butcher et al., 2003). The population density of the Eden Valley is as low as about 0.2 person/ha, lower than most districts in England. The major sources of income are agriculture, especially livestock rearing, tourism, and some industries (Butcher et al., 2003).

In the Eden Valley, the Permo-Triassic rocks lie in a fault-bounded basin bounded southwest by the Lake District and northeast by the North Pennines. As shown in Fig. 1, the principal aquifers in this region are the Penrith Sandstones and St Bees Sandstones, which are thick sequences of Permo-Triassic sandstones with moderate to high permeability and porosity. These sandstones are separated by the Eden Shale, an aquitard mainly composed of mudstone and siltstone. In the study area, approximately 75% of the sandstone aquifers are covered by superficial deposits, significantly impacting recharge and distribution (Allen et al., 2010). Hydraulic conductivity (K) ranges from 3.5 × 10^–5^ to 26.2 m/day for the Penrith Sandstones and from 0.048 to 3.5 m/day for St Bees Sandstones. The wide range is primarily due to the varying degree of cementation of the sandstone (Allen et al., 1997). Carboniferous limestone is mainly located on the edges of the study area, characterized by very low porosity and permeability. They provide base flow for the streams and tributaries of the catchment subregion of the River Eden.

The Eden Valley is largely rural and mainly covered by grassland, mountains, and arable land. It is a notable concern that intensive farming activities, including fertilizers and manure slurry applications, lead to groundwater nitrate contamination. According to the recent Nitrate Vulnerable Zones (NVZs) designation in 2021, there are four groundwater NVZs in the Eden Valley (EA, 2021). i.e., the Brampton Sand Sheet, Penrith, Skirwith, and Kirby Thore NVZs. Therefore, it is necessary to understand the nitrate contamination level in groundwater and analyze its key driving factors to tackle the nitrate challenge in the Eden Valley.

### Nitrate concentration data

Groundwater nitrate concentration data were collected from the Water Quality Archive (Beta), which was carried out by the EA (EA, 2012). In the Eden Valley, there are 1107 groundwater nitrate concentration measurements from 74 monitoring wells whose locations are shown in Fig. 1 between 2012 and 2021. 10.66% of nitrate values were below the method detection limit (0.196 mg/L-N), and they were set to half the limit (0.098 mg/L-N). For the well with multiple nitrate measurements in one year, the annual mean value was calculated to represent its average nitrate level in that year. Ultimately, 549 nitrate concentration data between 2012 and 2021 were used for training and testing the predictive model. In addition, to decrease the impact of very high values, nitrate concentrations were log_10_ transformed before modeling. The log_10_ transformed values represented the response variable for the machine learning models, and the predictions were then converted back to nitrate concentrations after modeling. Nitrate values in this study represent nitrate nitrogen, with the unit expressed as mg/L-N.

### Predictor variables and feature engineering

We compiled a set of 26 predictor variables that represented climate, hydrology, soils, geology, hydrogeology, and land use, as listed in Table S1. Superficial depth data was from British Geological Survey (BGS, 2020). Soil physical and chemical characteristics were obtained from the European Soil Data Centre (ESDAC) (Ballabio et al., 2016, 2019). The dataset of precipitation and evaporation was from the UK Met Office (Met Office et al., 2018). Furthermore, the baseflow index (BFI) (Boorman et al., 1995) and land use (Morton et al., 2014) were collected from the UK Centre for Ecology and Hydrology (CEH). In the Eden Valley, the main land use was grassland (58.90%), woodland (9.98%), arable land (9.71%), built-up areas (1.98%), and mountain (18.64%), respectively. The former four land use types were used to analyze the impacts on the groundwater nitrate in this study, and the contributing area was calculated within a 500 m radius circular buffer (Ransom et al., 2022). Moreover, some variables were obtained from the previous study (Wang & Burke, 2017), including elevation, groundwater average recharge, unsaturated zone thickness, and aquifer properties. Then, all of the environmental variables at the well locations and the center of each element in the grid map of the Eden Valley (200 m × 200 m), except for land use, were extracted as point data using ArcGIS.

**Fig. 2:**
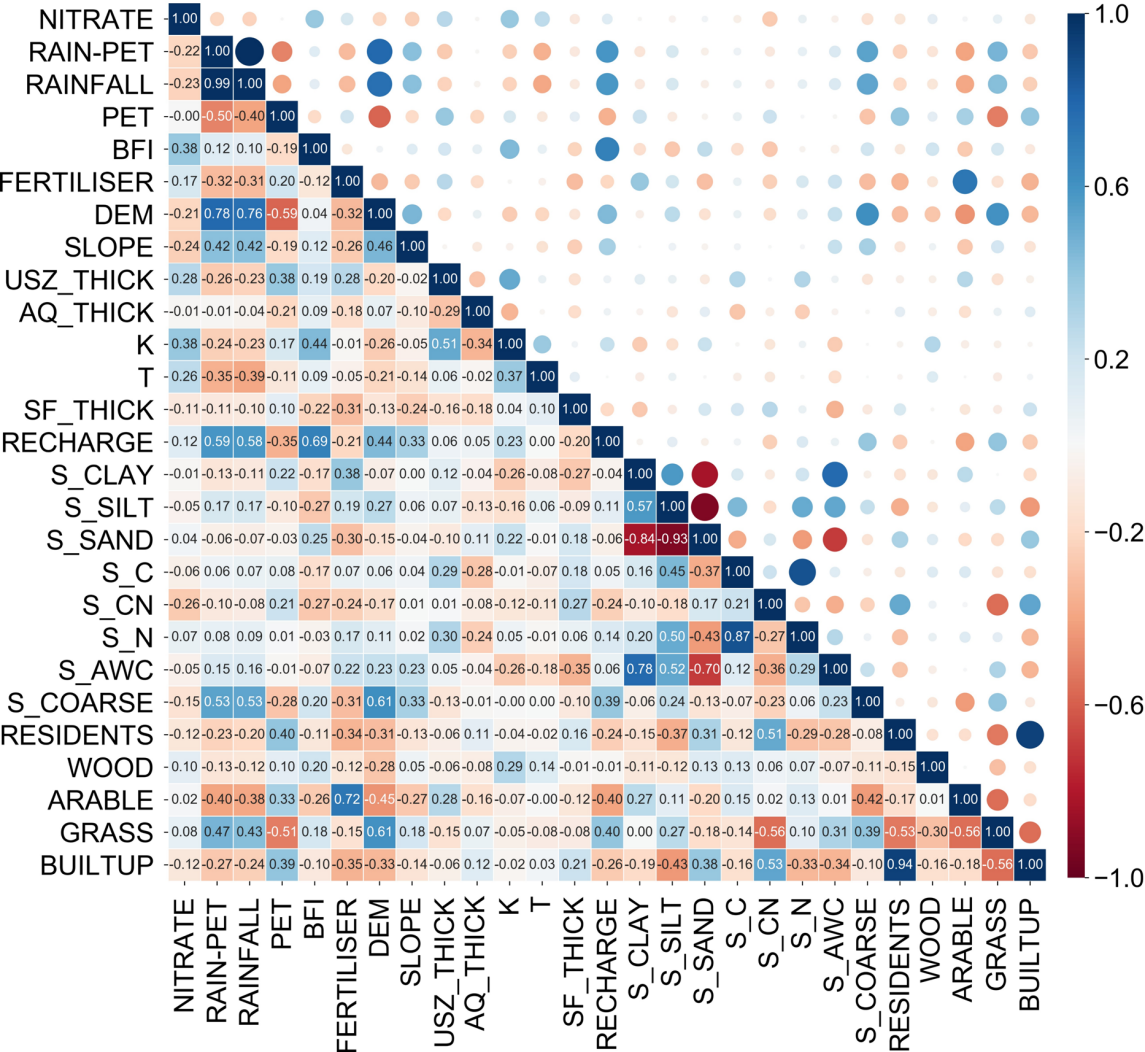
The heatmap of Pearson correlation matrix

To reduce multicollinearity in the dataset, prevent overfitting and enhance explanation, the Pearson correlation coefficient (r) between the environmental variables was calculated, as illustrated in the heatmap of correlation matrix (Fig. 2). Based on the absolute value of r exceeding 0.70, four highly correlated variables exhibiting a higher average absolute value of r with other variables were removed (Kuhn & Johnson, 2013), including precipitation minus evaporation, nitrogen fertilizer application rates, nitrogen in the soil, and available water capacity. Despite the average absolute correlation of the percentage of built-up area being greater than that of population, the great concern about the effects of land use on groundwater pollution led to the exclusion of the population. Similarly, soil sand percentage and DEM were also reserved, which are essential variables in nitrate predictions in previous research (Wheeler et al., 2015; Nolan et al., 2014). Eventually, 21 environmental variables were selected as input features for the ML models.

In addition, normalization was applied to ensure that each feature contributes equally to the result. It can help decrease the training time and improve the model performance. In this study, all the predictor variables were normalized to the range of 0 to 1 through min–max normalization before being utilized as inputs, as Eq. (1):

1 $$ X^{\prime} = \frac{{X - X_{\min } }}{{X_{\max } - X_{\min } }} $$

where $X^{\prime}$ represents the normalized value; $X$ is the original value, and ${X}_{max}$ and ${X}_{min}$ are the maximum and minimum of the original data, respectively.

### Interpretable stacking ensemble learning (ISEL) framework

**Fig. 3:**
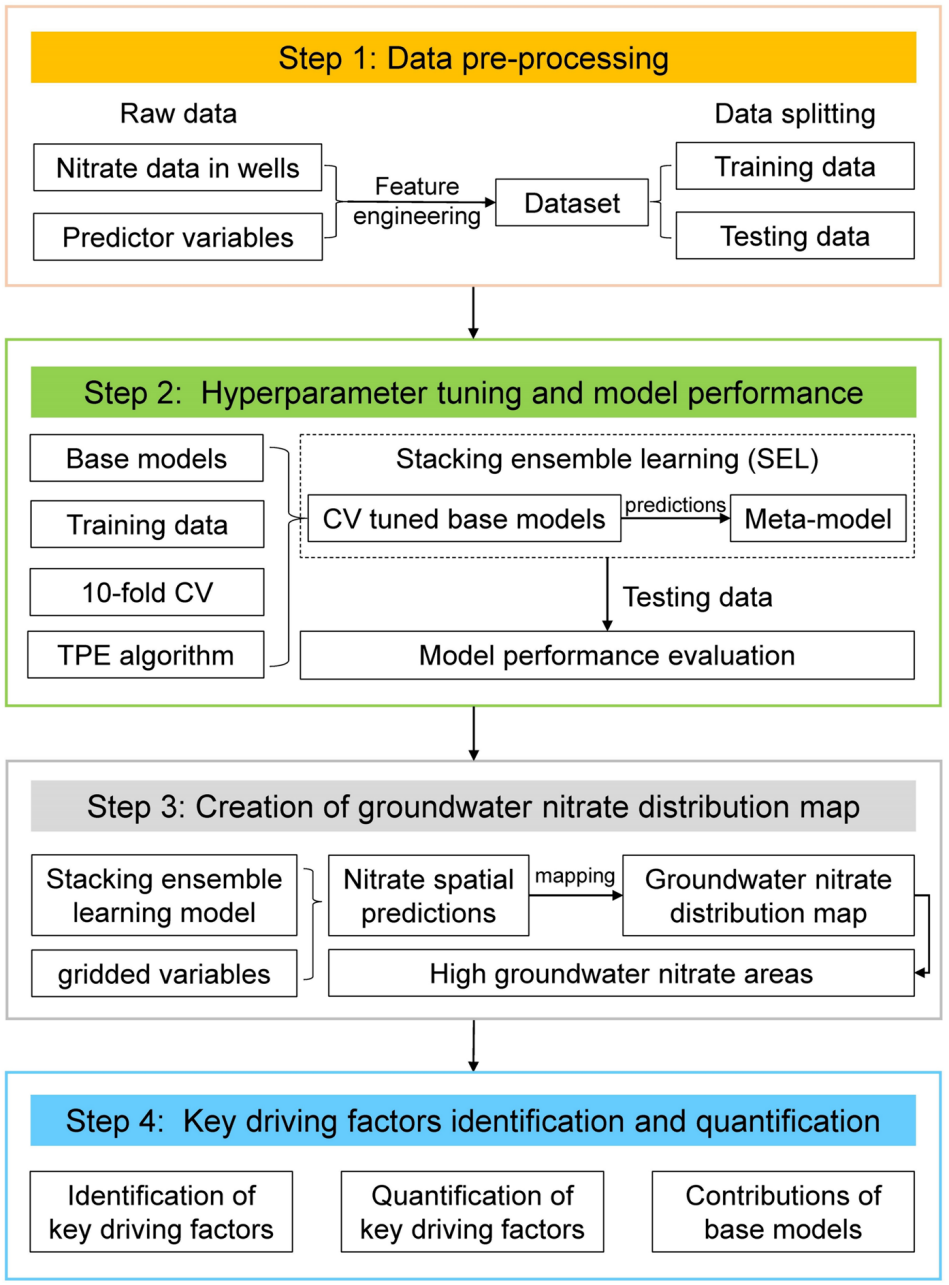
The framework of interpretable stacking ensemble learning (ISEL) for identifying the spatial distribution and driving factors of nitrate in groundwater

To improve the model performance and generalization and interpret the predictive model, we designed an ISEL framework, as shown in Fig. 3. The ISEL framework for groundwater nitrate mapping consists of four steps: (1) data pre-processing; (2) hyperparameter tuning and model performance evaluation; (3) creation of groundwater nitrate distribution map; and (4) key driving factors identification and quantitative analysis.

#### Stacking ensemble learning (SEL)

**Fig. 4:**
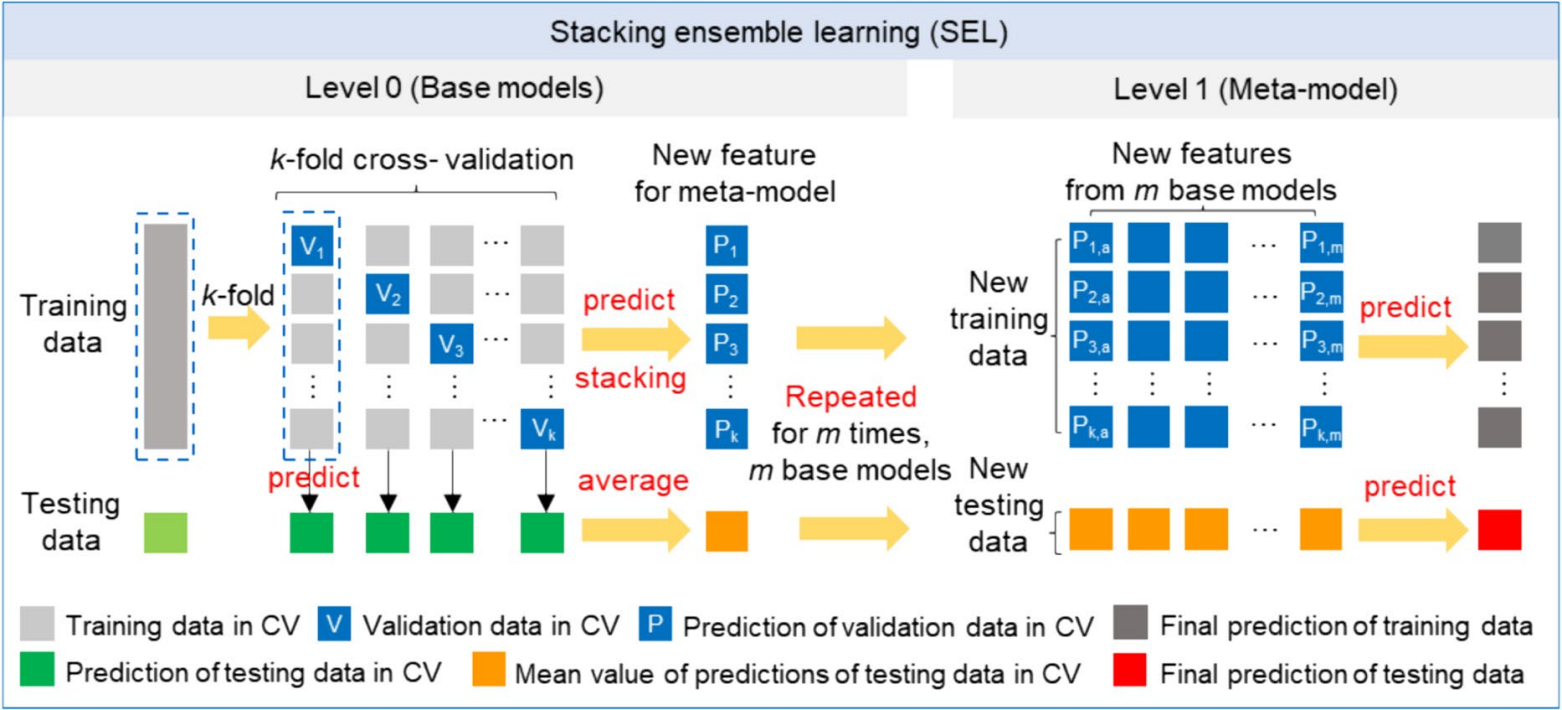
The workflow of the stacking ensemble learning (SEL) model

Stacking, also known as a stacked generalization, is a powerful ensemble learning technique in machine learning. It aims to improve predictive performance by relying on the “wisdom of the crowds”. The main idea of stacking is to extract more information from the base models, capture more complex patterns, and reduce the variance and bias of the individual models by integrating the predictions of multiple models. As a result, the SEL model typically performed better than the individual models because of the model diversity, bias reduction, and enhanced robustness. In the SEL model, the models in the first layer are trained on the original dataset, while the models in subsequent layers are trained on the outputs of the previous layer, as illustrated in Fig. 4.

In this study, we employed a two-level SEL model, consisting of five base models (GBDT, XGB, RF, ET, KNN) and a meta-model that uses the outputs from the base models. These models were selected because they are based on different theories and structures, are widely used, and have demonstrated high accuracy in previous studies. Moreover, the tenfold CV generator was applied in the training phase to improve model generalization capability. As shown in Fig. 4, the training data was divided into ten folds randomly; nine folds (in light grey) were used for training the models and one remaining fold (in dark blue) was reserved for validation in each iteration. By repeating this process ten times, we obtained ten predictive validation sets, which were then combined to form a new feature set for training the meta-model. Furthermore, at level 1, the average predictions (in orange) for the testing data from each iteration (in dark green) were used as a feature of new testing data for the meta-model. Consequently, the five base models provided five columns of new features as new training data and testing data for the meta-model. Finally, we can tune and fit the meta-model using new training data and evaluate model performance using new testing data.

To implement the methodology, we used the Scikit-Learn library (Pedregosa et al., 2011) in Python 3.7 (Van Rossum & Drake, 2009) for GBDT, RF, ET, KNN, and SEL. For the XGB model, the XGBoost package in Python (Chen & Guestrin, 2016) was applied.

#### Hyperparameter tuning

Following the commonly utilized 8:2 dataset splitting ratio (Joseph, 2022), ML models were developed using the training data from the first eight years (n = 472, 2012–2019), and the model performance was evaluated with the independent testing data from the subsequent two years (n = 77, 2020–2021). During model tuning, the optimal combination of hyperparameters was selected using the Tree-structured Parzen Estimator (TPE) algorithm (Bergstra et al., 2011) combined with the tenfold CV. TPE algorithm, a Bayesian optimization approach based on Gaussian mixture models, runs faster and performs more efficiently than Gaussian process models. It was conducted using the Python package Hyperopt (Bergstra et al., 2015). The initial range for the hyperparameter to be optimized was assigned according to relevant articles and documents, and the model was trained 1000 times to select the optimal combination of hyperparameters using the TPE algorithm. Moreover, tenfold CV technique was performed on the training data during model tuning to control model overfitting and enhance model generalizability.

After determining the optimal combination of hyperparameters, the whole training data was utilized to refit the CV-tuned model, and the testing data was then used to predict and compare model performance. Therefore, nitrate spatial predictions can be produced based on the 21 predictor variables and the CV-tuned model using Python. Finally, model predictions for mapping the nitrate spatial distribution in groundwater were performed using ArcGIS.

#### Model performance evaluation metrics

Three evaluation metrics were utilized to compare the predictive performance of different machine learning models: mean absolute error (MAE), root mean squared error (RMSE), and coefficient of determination (R^2^). MAE and RMSE reflect the average absolute difference and the average distance between the nitrate predictions and observations, respectively, as presented in Eqs. (2) and (3). R^2^ indicates the proportion of variance in the target variable that can be explained by the predictor variables, calculated as Eq. (4). Moreover, the mean R^2^ of tenfold CV was used to evaluate model generalization.

2 $$ MAE = \frac{{\mathop \sum \nolimits_{i = 1}^{n} \left| {\widehat{{y_{i} }} - y_{i} } \right| }}{n} $$

3 $$ RMSE = \sqrt {\frac{{\mathop \sum \nolimits_{i = 1}^{n} \left( {\widehat{{y_{i} }} - y_{i} } \right)^{2} }}{n} } $$

4 $$ R^{2} = 1 - \frac{{\mathop \sum \nolimits_{i = 1}^{n} \left( {\widehat{{y_{i} }} - y_{i} } \right)^{2} }}{{\mathop \sum \nolimits_{i = 1}^{n} \left( {\overline{{y_{i} }} - y_{i} } \right)^{2} }} $$

where $y_{i}$ is the $i^{th}$ observed value; $\widehat{{y_{i} }}$ is the $i^{th}$ predicted value;$\overline{{y_{i} }}$ is the mean value of the observed values; $n$ is the number of samples.

#### Model interpretability

SHAP is a recently developed unified measure of feature importance, which can help to improve the understanding of the predictions made by ML models (Lundberg & Lee, 2017)**.** It is based on game theory and uses an additive feature attribution method where the model output is a linear combination of input variables. The SHAP value represents the marginal contribution of each feature to each prediction (Lundberg et al., 2020). Compared to previous feature importance methods, SHAP provides richer explanations that interpret models locally and globally, and the global explanations are built according to local explanations, ensuring consistency. It can also identify whether the contribution of each input feature is positive or negative based on SHAP values.

The SHAP method was applied in this study to analyze the local and global feature importance to understand the importance and influence of driving factors on groundwater nitrate spatial predictions, as well as model contributions from base models to the meta-model. The SHAP analysis was implemented using the Python package SHAP (Lundberg & Lee, 2017).

## Results and discussion

### Groundwater nitrate data summary

As shown in Fig. S2, for the whole dataset (n = 549), the annual average groundwater nitrate concentrations ranged from 0.098 to 52.06 mg/L-N from 2012 to 2021, with a mean concentration and a standard deviation of 6.31 mg/L-N and 6.70 mg/L-N, respectively. The 25th, 50th, and 75th percentile groundwater nitrate concentrations were 0.94, 4.41, and 9.87 mg/L-N, respectively. Overall, 20.79% of the samples exhibited high nitrate concentrations, exceeding the maximum admissible concentration (MAC) of nitrate in water for human consumption (11.3 mg/L-N), as set by the European Union (EU) in the Drinking Water Directive 80/778/EEC. These high nitrate concentrations were mainly located in St Bees Sandstones and Penrith Sandstones, the central part of the Eden Valley. The percentage of wells with groundwater nitrate below 2 mg/L-N was the largest (37.16%). These wells were concentrated in the limestone and north of the St Bees Sandstones, the catchment subregion throughout the Eden Valley.

The whole nitrate concentration data between 2012 and 2021 (n = 549) was divided into a training set (n = 472, 2012–2019) and a testing data set (n = 77, 2020–2021), as shown in Fig. S2. Training data ranged from 0.098 to 52.06 mg/L-N, and testing data ranged from 0.098 to 30.00 mg/L-N. Moreover, the first, second, and third quartiles of training data are 0.94, 4.44, and 9.63 mg/L-N, respectively, which are 1.00, 4.20, and 11.00 mg/L-N for testing data. In general, the distributions of the training and testing datasets were similar, which may help mitigate the tendency for the method to overfit the training data.

### Hyperparameter tuning and model performance

The optimal hyperparameters of ML models were determined using the TPE optimization algorithm combined with the maximum tenfold CV mean R^2^ criterion by training 1000 times (Table S2). Model performance was compared according to the evaluation metrics for the testing data: MAE, RMSE, and R^2^ (Table 1). All individual and SEL models produced satisfying predictions and were considered acceptable. Based on the testing R^2^, the model performance ranked in the following order: SEL > GBDT > XGB > RF > ET > KNN. Compared to the five individual models, the SEL model had the lowest MAE (0.1229) and RMSE (0.2586) and the highest R^2^ (0.8644) for testing data, which indicated that the SEL model outperformed the other five individual models in predictive performance. Furthermore, in terms of generalization ability, models ranked the same as the model performance based on the mean R^2^ of tenfold CV. The SEL model had the highest CV mean R^2^ of 0.8500, which was 2.68–4.90% higher than the other models, and the smallest CV standard deviation of 0.0702, suggesting better generalization and stability. Thus, in contrast with the five individual models, the two-level heterogeneous SEL model enhanced predictive performance and generalization ability.

**Table 1:** Model performance metrics for the models: gradient boosting decision tree (GBDT), extreme gradient boosting (XGB), random forest (RF), extremely randomized trees (ET), k-nearest neighbors (KNN), and stacking ensemble learning (SEL)

Model	Tenfold CV R^2^ (mean ± std.)	Training data (n = 472)			Testing data (n = 77)		
		MAE	RMSE	R^2^	MAE	RMSE	R^2^
GBDT	0.8416 ± 0.0971	0.0999	0.2072	0.9000	0.1254	0.2618	0.8610
XGB	0.8400 ± 0.1082	0.0997	0.2056	0.9016	0.1263	0.2651	0.8575
RF	0.8368 ± 0.0910	0.1023	0.2114	0.8960	0.1271	0.2680	0.8544
ET	0.8363 ± 0.0954	0.1060	0.2140	0.8934	0.1315	0.2763	0.8452
KNN	0.8240 ± 0.1392	0.0958	0.2078	0.8994	0.1283	0.2859	0.8342
SEL	0.8500 ± 0.0702	0.1037	0.2112	0.8961	**0.1229**	**0.2586**	**0.8644**

The units of MAE and RMSE are log_10_ (mg/L-N), and std. represents standard deviation. Bold text indicates the best performance according to the evaluation metric

**Fig. 5:**
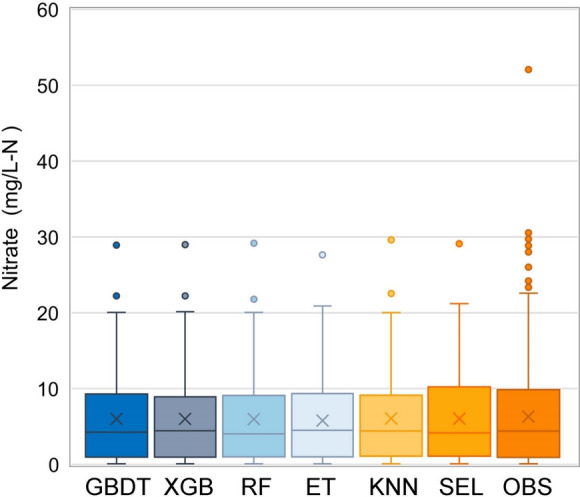
The box plots of observed (OBS) and predicted groundwater nitrate concentrations from the models: gradient boosting decision tree (GBDT), extreme gradient boosting (XGB), random forest (RF), extremely randomized trees (ET), k-nearest neighbors (KNN), and stacking ensemble learning (SEL)

The box plots of predicted and observed groundwater nitrate concentrations were displayed in Fig. 5, visually representing the spread of nitrate values. To contrast the predicted and observed nitrate concentrations, we retransformed the predicted values back to nitrate concentrations. In Fig. 5, the lower and upper ends of the box denote the 25th and 75th percentiles (${Q}_{1}$ and ${Q}_{3}$), the horizontal line inside the box represents the 50th percentile (the median), and the cross indicates the mean value. Moreover, the lower whisker represents the minimum nitrate value, and the upper whisker denotes the value of ${Q}_{3}+1.5{{(Q}_{3}-Q}_{1})$, excluding the outliers that drawn as points.

In Fig. 5, it can be observed that the minimum (0.10 mg/L-N) and the first quartile (0.96–1.11 mg/L-N) of nitrate predictions from all models were similar to those of the observation (0.10 mg/L-N, 0.94 mg/L-N). Whereas the third quartile (8.91–9.35 mg/L-N) and the upper whisper (20.03–20.15 mg/L-N) from the five individual models were apparently lower than those of the SEL model (10.24 mg/L-N, 21.22 mg/L-N) and observation (9.94 mg/L-N, 23.35 mg/L-N), indicating that the predictions for five individual models were biased in high values. By contrast, the SEL model had a more reliable range of groundwater nitrate predictions, closer to the observations than the other five individual models. Moreover, the mean value of nitrate predictions from the SEL model (5.60 mg/L-N) was comparable to the observation (5.65 mg/L-N), which is marked by a cross in Fig. 5. In comparison, the mean values of the predictions from the individual models were 5.33–5.42 mg/L-N, suggesting that their predicted results were generally lower than the observed values. Furthermore, the standard deviation of predictions from the SEL model (5.34 mg/L-N) was also quite close to the observations (5.40 mg/L-N), revealing that its predictions were dispersed similarly to the observation. Overall, the distribution of nitrate predictions from the SEL model was comparable to that of the observations at the training and testing phases in terms of the range, mean value, and standard deviation.

From the analysis above, it can be concluded that the SEL model exhibited superior predictive performance and generalization, indicating that its nitrate predictions were more reliable. Although GBDT and XGB performed relatively well, their high nitrate predictions were obviously lower than those of the SEL model and observations. This is probably because the ensemble tree regression models typically reduce the variance of predictions but leave bias, resulting in negative and positive bias for big and small values, respectively (Belitz & Stackelberg, 2021; Zhang & Lu, 2012). Thus, the SEL model can be a powerful tool for accurately predicting groundwater nitrate concentrations at unsampled locations.

### Nitrate predictions and spatial distribution

After the training and testing phases, the SEL model was applied to predict groundwater nitrate concentrations across the 200 m × 200 m grid map covering the Eden Valley using environmental variables. Table 2 summarizes the percentages of different concentration ranges of groundwater nitrate spatial predictions for the SEL model. According to the statistical metrics, the predicted nitrate concentrations across the Eden Valley ranged from 0.11 to 27.27 mg/L-N, consistent with the observations excluding the outliers. The median and mean values for nitrate spatial predictions were 1.10 and 2.22 mg/L-N, respectively, indicating that nitrate concentrations are generally low at most locations in the study area. As shown in Table 2, the percentage of nitrate concentration classes decreased as the concentration increased. The predicted nitrate concentrations in the range of 0–2 mg/L-N accounted for the largest proportion at 67.36%, followed by the 2–5 mg/L-N (16.78%), 5–8 mg/L-N (10.85%), and 8–11.3 mg/L-N (4.22%) classes, respectively. By contrast, the areas with high groundwater nitrate concentrations exceeding the MAC of 11.3 mg/L-N only occupied 0.79% of the total, the lowest proportion within the study area, and these areas accounted for 2.46% of the sandstone aquifers.

**Table 2:** Percentages of different ranges of groundwater nitrate spatial predictions in the Eden Valley, utilizing the stacking ensemble learning (SEL) model

Nitrate (mg/L-N)	0–2	2–5	5–8	8–11.3	≥ 11.3
Percentage (%)	67.36	16.78	10.85	4.22	0.79

**Fig. 6:**
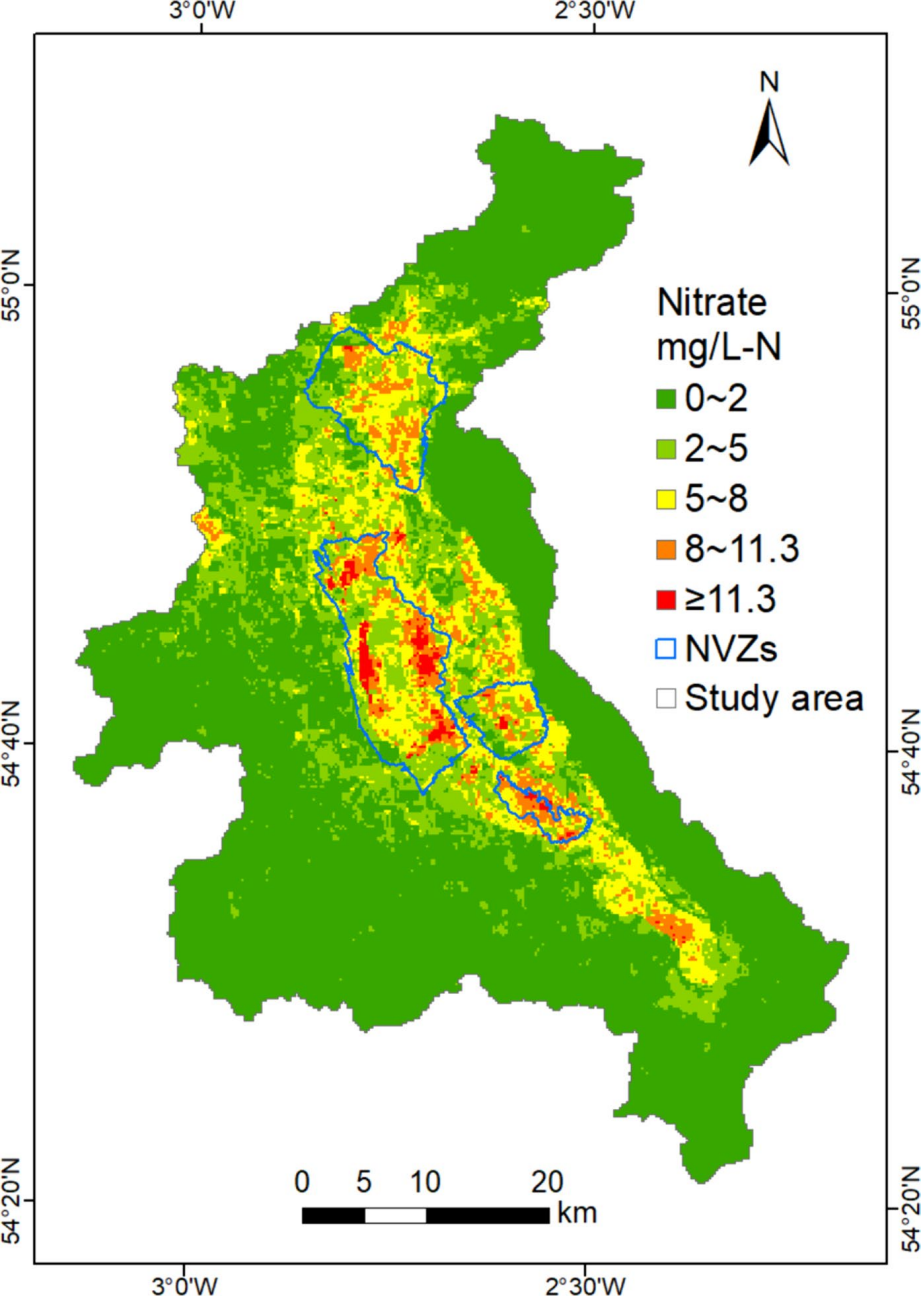
Spatial distribution of predicted nitrate concentrations in groundwater for the SEL model at 200 m × 200 m resolution in the Eden Valley

Figure 6 shows the 200 m × 200 m spatial distribution grid map of predicted groundwater nitrate concentrations for the SEL model in the Eden Valley, representing the average annual nitrate level between 2012 and 2021. The results suggested that its distribution pattern is similar to the nitrate input reported in the previous study (Wang & Burke, 2017). Moreover, nearly 91.26% of high nitrate predictions exceeding 11.3 mg/L-N are located inside the NVZs, revealing that the predicted spatial distribution of groundwater nitrate for the SEL model is reliable. As illustrated in Fig. 6, predicted groundwater nitrate concentrations in most of the central part of the valley, were generally above 2 mg/L-N, whereas concentrations in other aquifers were predominantly below 2 mg/L-N. Furthermore, the high nitrate concentrations exceeding 11.3 mg/L-N were concentrated in the Penrith Sandstone aquifer where arable land and grassland predominated. It is evident that the groundwater nitrate contamination is primarily attributed to agriculture in the study area, which is in line with earlier investigations (Allen et al., 1997; Butcher et al., 2003). Therefore, it is necessary to control the application of N-fertilizers and animal manure to reduce nitrogen pollution sources in high groundwater nitrate areas and surrounding regions, as required by the NVZ regulations (EU, 1991). In addition, drip irrigation is suggested as a substitute for flood irrigation to limit nitrogen leaching from the bottom of the soil.

According to the nitrate spatial predictions from the SEL model, it is worth noting that about 8.74% of the high groundwater nitrate areas are located outside the designed NVZs. These areas are concentrated in the southeast and northeast of the Penrith NVZ, as well as the southeast of the Kirby Thore NVZ, and have the potential to exacerbate groundwater nitrate contamination without any mitigative measures. Based on the previous study (Wang & Burke, 2017), they are areas with high to moderate high nitrogen input. Thus, it is necessary to consider delineating these areas into the NVZs in the future and formulate targeted management strategies. Moreover, a small portion of built-up areas in the central part of the valley are quite close to high nitrate locations. Hence, water managers should be cautious about potential health issues when directly using local groundwater.

### Quantitative analysis of driving factors and base models

#### Contributions of driving factors to nitrate predictions

**Fig. 7:**
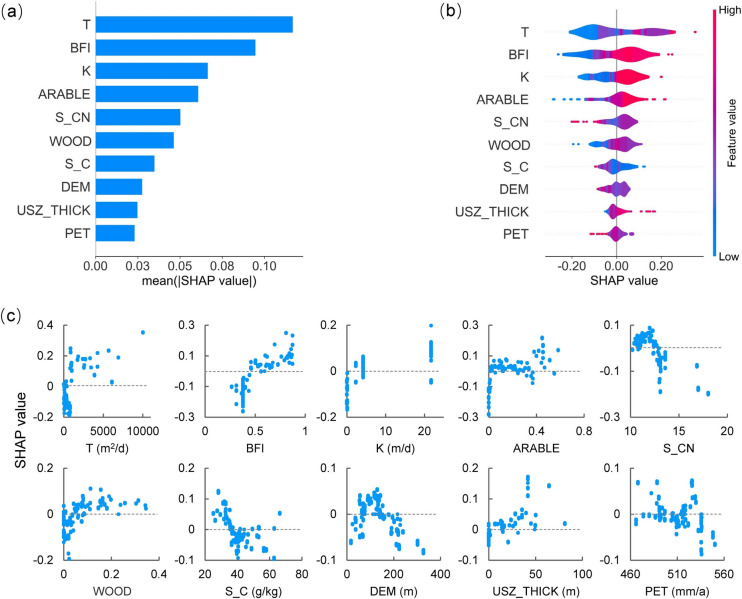
SHAP analysis for training data. **a** The average absolute value of SHAP values, **b** SHAP values, and **c** SHAP dependence plots of the top ten essential variables for the stacking ensemble learning (SEL) model

The importance and influence of the driving factors underlying the nitrate predictions on the training data were quantitatively analyzed using the SHAP method, offering valuable insights into the relationship between environmental variables and groundwater nitrate concentrations. Figure 7a illustrates the global variable importance ranking based on the mean absolute value of SHAP values shown on the x-axis, denoting the average impact on model output magnitude. Figure 7b presents the SHAP summary plot as a violin plot, illustrating the global distribution of feature influence. The y-axis lists the top ten most important variables, and the x-axis represents the SHAP value of each instance for the feature. Moreover, the width of the violin plot denotes the frequency of the SHAP value, and the color indicates the average feature value at that position, with red and blue signifying high and low relative values of the variables, respectively. Figure 7c displays the local SHAP values for each value of the top ten crucial driving factors and shows the relationship between the environmental variables (x-axis) and SHAP values (y-axis), providing insights into how nitrate predictions vary with the increasing values of the variables.

As shown in Fig. 7a, the top ten crucial variables for the SEL model can be generally categorized into the following five categories: hydrogeology, hydrology, land use, soil organic matter, and topography. Transmissivity (T) and K are essential hydrogeological parameters representing the ability of an aquifer to transmit and conduct water, both of which are related to groundwater flow rate (Wang et al., 2013). They are the most and the third-most important driving factors on groundwater nitrate predictions for the SEL model, respectively, and both have a positive impact (Fig. 7b and c), consistent with the finding of earlier research (Wang & Burke, 2017). High T and K can accelerate groundwater flow, thereby facilitating the migration and dispersion of nitrate (Jang et al., 2017). Moreover, rapid groundwater flow can reduce the potential for nitrate to interact with microorganisms and other substances, hindering denitrification processes and preventing effective nitrate removal (Rivett et al., 2008). This ultimately increases the risk of nitrate pollution in groundwater (Aller et al., 1987).

BFI is a critical index that reflects the contribution of groundwater to river flow. It emerged as the second most important variable and exhibited a positive correlation with nitrate predictions. This can be attributed to the fact that BFI is positively correlated with groundwater recharge (r = 0.69) (Zomlot et al., 2015). A higher BFI signifies greater recharge, which can enhance the transport of nitrogen from the surface to the aquifer and facilitate nitrate leaching into groundwater. It can potentially raise groundwater nitrate levels (Nolan and Hitt, 2006), particularly in areas with high agricultural nitrogen loading (Böhlke, 2002). Although increased recharge can contribute to the dilution of groundwater nitrate, in agriculturally intensive areas such as the Eden Valley, this effect is likely less significant than the substantial nitrate leaching into the groundwater. In contrast, potential evapotranspiration (PET) showed a negative correlation with recharge (r = -0.35) (Walker et al., 2019). Therefore, increased PET suggests reduced recharge, which may limit contaminants leaching into the aquifer, resulting in lower nitrate levels in groundwater.

Furthermore, the percentage of arable land and woodland within a 500 m radius circular buffer ranked fourth and sixth in the SEL model, respectively, and were associated with high nitrate concentrations, as shown in Fig. 7c. The possible reason is that the arable land percentage and fertilizer application rate are highly correlated (r = 0.72) (Fig. 2), in line with the previous findings (Butcher et al., 2003; Ransom et al., 2022). Extensive fertilizer and manure utilization in arable land can enhance crop growth and promote nitrification (Zhang et al., 2013). Thus, excessive nitrogen unabsorbed by crops likely leads to an elevated nitrate level. Furthermore, the positive influence of woodland on elevated groundwater nitrate levels is possibly due to abundant nitrogen from various sources, such as atmospheric nitrogen deposition, litter decomposition, and biological nitrogen fixation (Sardar et al., 2023). Notably, atmospheric nitrogen deposition in most woodlands in the UK surpasses the critical loads (Vanguelova et al., 2024), enhancing nitrogen mineralization and nitrification in the soil (Zhu et al., 2015), thereby raising the likelihood of nitrate leaching into groundwater (Dise & Wright, 1995).

Conversely, groundwater nitrate concentrations tended to decrease with increasing C:N ratio and organic carbon content in the soil, which ranked fifth and seventh in importance. Elevated C:N ratios and soil organic carbon can restrict the availability of nitrogen sources essential for microbial metabolism (Hoang et al., 2022). It has been reported that a high C:N ratio in soil adversely impacts ammonifying bacteria, facilitating soil organic nitrogen conversion into ammonium nitrogen (Yang et al., 2023). The nitrification process is closely related to the ammonium nitrogen production rate (Booth et al., 2005), and thus, insufficient nitrogen can significantly hamper the nitrification process. In addition, an abundance of organic carbon in soil can strengthen the activity of denitrifying bacteria, which are mostly facultative anaerobic heterotrophs, favoring denitrification and reducing nitrate levels (Sheng et al., 2018). Consequently, a high C:N ratio and increased organic carbon content can help prevent nitrate accumulation in soil and reduce nitrate leaching losses (Bai et al., 2021), thereby decreasing the risk of nitrate pollution in groundwater.

Moreover, elevation was ranked as the eighth most significant influencing factor. As shown in Fig. 7c, the SHAP value implied a positive correlation with elevation, peaking at around 130 m before gradually decreasing. Specifically, 86.4% of the samples with positive SHAP values fall within the elevation range of 60–150 m, where the positive influence on high nitrate predictions is stronger than the negative, as illustrated in Fig. S3a. These elevations are predominantly located along the River Eden (Fig. S2), which is suitable for farming. Fig. S3b reveals that when the percentage of arable land exceeds 5%, 72.8% of the samples are situated at an elevation ranging from 60 to 150 m, holding a significantly larger proportion of samples compared to other elevation intervals. Therefore, prevalent agricultural practices on arable land at these elevations, including the applications of chemical fertilizers and manure, likely contribute to the elevated groundwater nitrate level.

In addition, it should be noted that a thicker unsaturated zone is associated with higher groundwater nitrate concentrations (Böhlke, 2002) This is probably because of the longer lag time for peak nitrate leaching in the 1980s in areas with a thick unsaturated zone, which has arrived at groundwater table in the 1990s in regions with a thinner unsaturated zone (Wang et al., 2013). Furthermore, due to limited data access, this study used long-term average values for the unsaturated zone thickness. If data on the temporal dynamics of the unsaturated zone thickness or groundwater table become available, further research could explore their impacts on nitrate concentrations in groundwater.

#### Contributions of base models to the meta-model

In the stacking model, the output from the base model was used as the input for the meta-model. To assess the contribution of base models to the meta-model, the importance of the base model was analyzed by employing SHAP. Based on the mean absolute value of SHAP values, the five base models at level 0 exhibited positive impacts on the meta-model at level 1, with the following ranking: XGB > KNN > GBDT > RF > ET.

In the SEL model, the average absolute values of SHAP values of the outputs from both XGB and KNN were nearly 0.12, higher than those of the other base models. It is likely because that the importance rankings of the percentage of woodland in a 500 m radius circular buffer in the XGB and KNN are higher (the third) compared to other base models (the fifth or sixth), as shown in Fig. S4b and e. Conversely, the average absolute value of SHAP values of the output from the ET model was below 0.10, which was obviously lower than those of the other base models. This may be associated with the percentage of arable land, which ranked tenth in the ET model (Fig. S4d) but in the top five in the other four base models and in the SEL model.

Furthermore, in the top three performing models (i.e., GBDT, XGB, and RF), T was identified as the most influential variable (Fig. S4a–c). Another variable related to aquifer characteristics, K, ranked in the top five in four of the base models, excluding the GBDT.

In conclusion, the contribution analysis of driving factors to the final nitrate predictions, as well as the impacts of the base models, suggests that the effects of hydrogeology, hydrology, land use, soil organic matter, and elevation in this study are consistent with previous findings (Aller et al., 1987; Butcher et al., 2003). The results reveal that hydrogeological conditions (T and K) and land use (particularly arable land and woodland) play a crucial role in predicting groundwater nitrate concentrations in the Eden Valley. Consequently, from the perspective of genesis analysis, nitrate spatial predictions from the SEL model are reliable. It is essential for water environment managers to formulate targeted strategies to manage fertilizer application and manure storage, especially in areas with high nitrogen loading and fast groundwater flow.

## Conclusions

Nitrate is a widespread pollutant in groundwater, threatening human health and environmental safety worldwide. This study developed a novel framework for identifying the spatial pattern of groundwater nitrate concentration with high accuracy and quantitatively analyzing the importance of key driving factors. The results demonstrate that the proposed ISEL framework is effective in the Eden Valley. The SEL model improved predictive performance and generalization ability compared to the five individual ML models (GBDT, XGB, RF, ET, KNN), providing reliable nitrate predictions. It was found that groundwater nitrate concentrations in 2.46% of sandstone aquifers exceed the MAC of 11.3 mg/L-N, while 8.74% of areas with high nitrate concentrations have not been delineated as the NVZs. SHAP analysis further reveals that groundwater nitrate levels are significantly affected by aquifer characteristics, and land use, with T identified as the most important factor in the SEL model. These findings can assist water environmental managers in developing targeted pollution control strategies to ensure sustainable groundwater quality management. This study marks the first integration of the stacking technique with an interpretability approach in the field of groundwater contaminant. Future research directions include predicting contaminant distribution across different spatial scales, modeling the spatiotemporal dynamics of pollutants and incorporating broader data sources such as remote sensing. Overall, the proposed framework offers a promising way to accurately predicting contaminants distribution and clarifying complex environmental phenomena, thereby contributing to sustainable development.

## Supplementary Information

Below is the link to the electronic supplementary material.

Supplementary file1 (DOCX 2270 KB)

## Data Availability

No datasets were generated or analysed during the current study.
