# Supplementary material for: Identifying the spatial pattern and driving factors of nitrate in groundwater using a novel framework of interpretable stacking ensemble learning
Source: Environ Geochem Health. 2024 Oct 29;46(11):482. doi: 10.1007/s10653-024-02201-1 (PMC11522174; doi:10.1007/s10653-024-02201-1)
Supplement: Supplementary file 1 — Supplementary file1 (DOCX 2270 KB) [file 10653_2024_2201_MOESM1_ESM.docx]

# Identifying the spatial pattern and driving factors of nitrate in groundwater using a novel framework of interpretable stacking ensemble learning

Xuan Li^a,b^, Guohua Liang^a^, Lei Wang^b^*, Yuesuo Yang^c^, Yuanyin Li^b,e^, Zhongguo Li^d^, Bin He^a^, Guoli Wang^a^.

^a^ *School of Hydraulic Engineering, Dalian University of Technology, Dalian, 116024, China.*

^b^ *British Geological Survey, Keyworth, Nottingham, NG12 5GG, UK.*

^c^ *Key Laboratory of Groundwater Resources and Environment, Jilin University, Ministry of Education, Changchun, 130021, China.*

^d^ *Liaoning Water Affairs Service Center, Shenyang, 110003, China.*

^e^ *Department of Geography, Durham University, Durham, DH1 3LE, UK.*

Corresponding author: L.Wang ([lelei@bgs.ac.uk](mailto:lelei@bgs.ac.uk))

**Appendix A. Supplementary data**

**Table S1**

Summary of predictor variables.

| **Variable** | **Description** | **Source** |
| --- | --- | --- |
| **Topography** | |  |
| DEM | Land surface elevation, m | (Wang & Burke, 2017) |
| SLOPE | Average slope, degree | Derived from DEM |
| **Climate** | |  |
| RAINFALL | Average precipitation, 2012- 2021, mm/a | (Met Office et al., 2018) |
| PET | Average potential evapotranspiration, 2012- 2021, mm/a |  |
| **Hydrology** |  |  |
| BFI | Baseflow index | (Boorman et al., 1995) |
| RECHARGE | Average recharge for the period 1961-2011, mm/d | (Wang & Burke, 2017) |
| **Land use** | |  |
| WOOD | Percentage of woodland in a 500m radius circular buffer, % | (Morton et al., 2014) |
| ARABLE | Percentage of arable land in the buffer, % |  |
| GRASS | Percentage of grassland in the buffer, % |  |
| BUILTUP | Percentage of built-up areas in the buffer, % |  |
| **Soil property** | |  |
| S_SAND | Sand content, % | (Ballabio et al., 2016) |
| S_SILT | Silt content, % |  |
| S_CLAY | Clay content, % |  |
| S_COARSE | Coarse fragments, % |  |
| S_CN | Carbon to nitrogen ratio |  |
| S_C | Organic carbon content of soils, g/kg |  |
| S_N | Organic nitrogen content of soils, g/kg |  |
| S_AWC | Available water capacity |  |
| **Geology** |  |  |
| SF_THICK | Superficial thickness, m | (BGS, 2020) |
| **Hydrogeology** | |  |
| AQ_THICK | Aquifer thickness, m | (Wang & Burke, 2017) |
| USZ_THICK | Unsaturated zone thickness, m |  |
| T | Transmissivity, m^2^/day |  |
| K | Average horizontal hydraulic conductivity, m/d |  |
| **Others** | |  |
| FERTILISER | Average annual nitrogen fertiliser application rate, kg/km^2^ | (Osório et al., 2019) |
| RESIDENTS | UK gridded population, 2011 | (Reis et al., 2017) |

**Table S2**

Optimal hyperparameters of the machine learning models, gradient boosting decision tree (GBDT), extreme gradient boosting (XGB), random forest (RF), extremely randomized trees (ET), and k-nearest neighbors (KNN) in level 0, and the meta-model, KNN, in level 1.

| Model | | Hyperparameters |
| --- | --- | --- |
| Base models | GBDT | n_estimators=185, learning_rate=0.03, max_depth=11, min_samples_leaf=13, subsample=1.0 |
|  | XGB | n_estimators=359, learning_rate=0.35, max_depth=13, min_child_weight=13, gamma=0.03, colsample_bytree =0.53, subsample=0.74, reg_lambda=5.84, reg_alpha=1.46 |
|  | RF | n_estimators=114, max_depth=18, min_samples_leaf=3, min_samples_split=5, max_features=20 |
|  | ET | criterion=‘mse’, n_estimators=157, min_samples_leaf=1, min_samples_split=6, max_depth=8 |
|  | KNN | weights=’distance’, n_neighbors=6 |
| Meta-model | KNN | weights=’distance’, n_neighbors=14 |


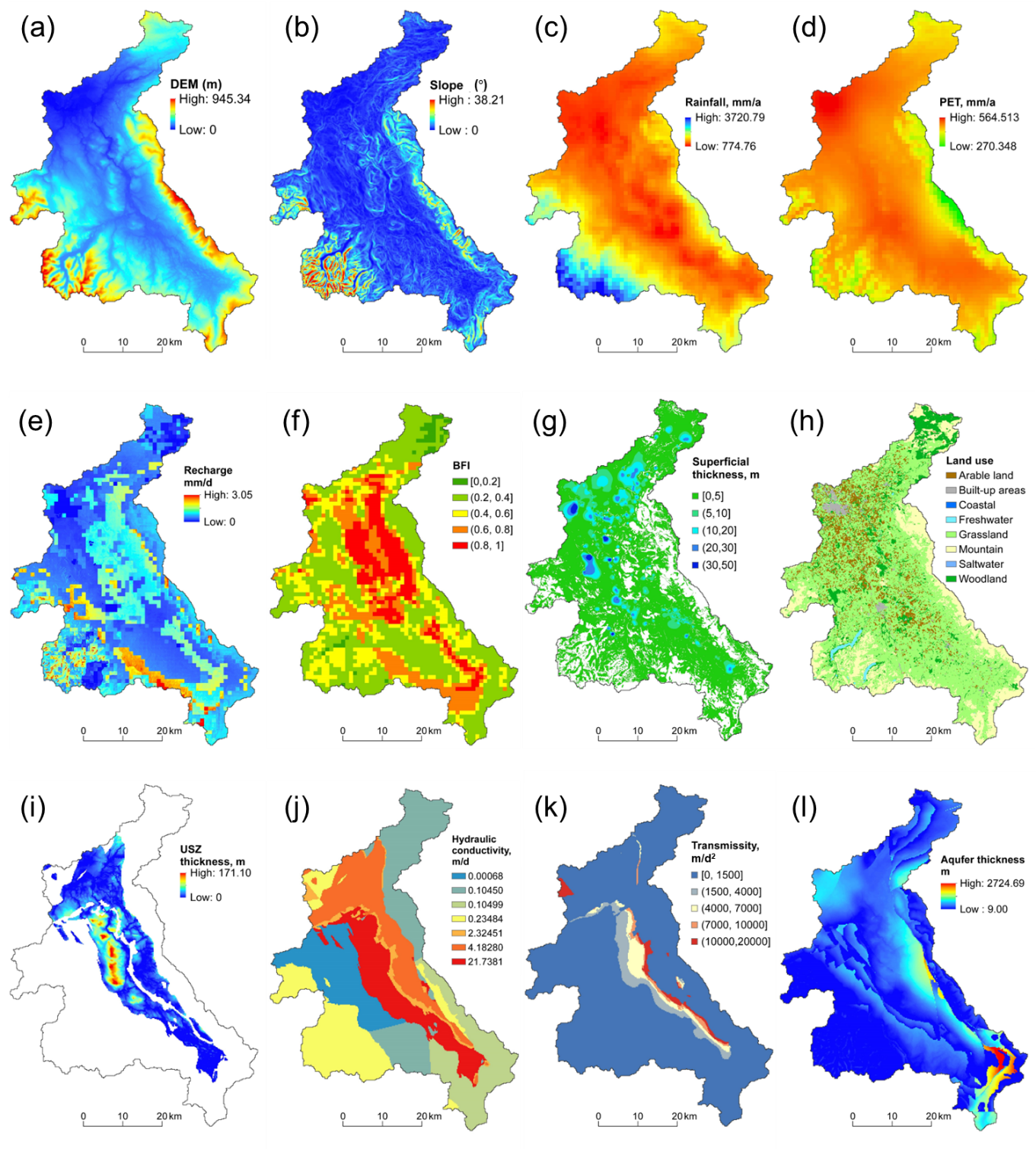


**Fig. S1.** Environmental variables in the Eden Valley.


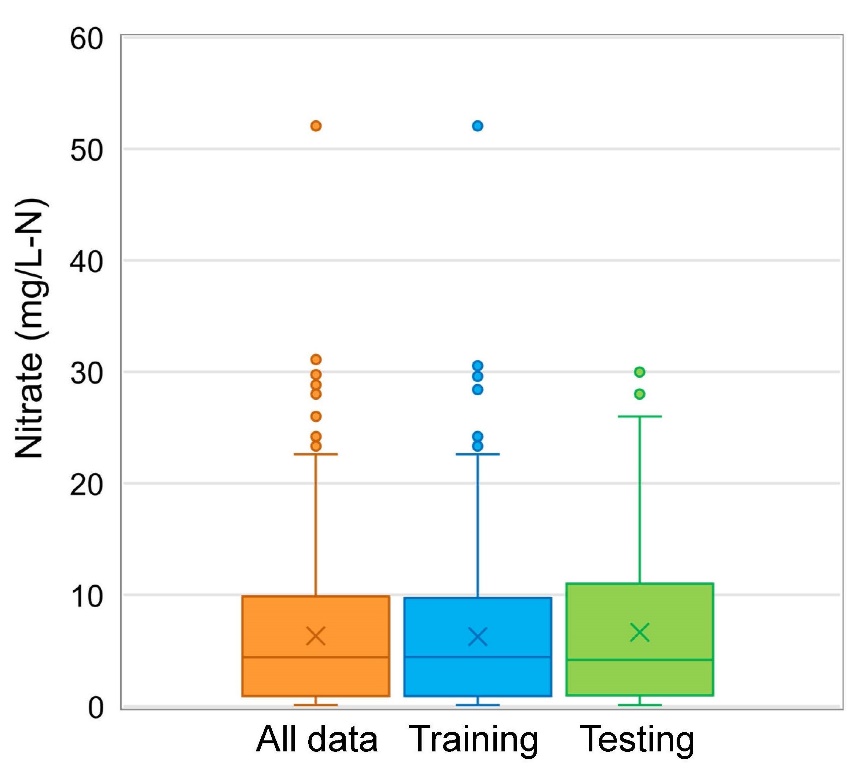


**Fig. S2.** The boxplot of groundwater nitrate concentrations for all data, training data, and testing data.


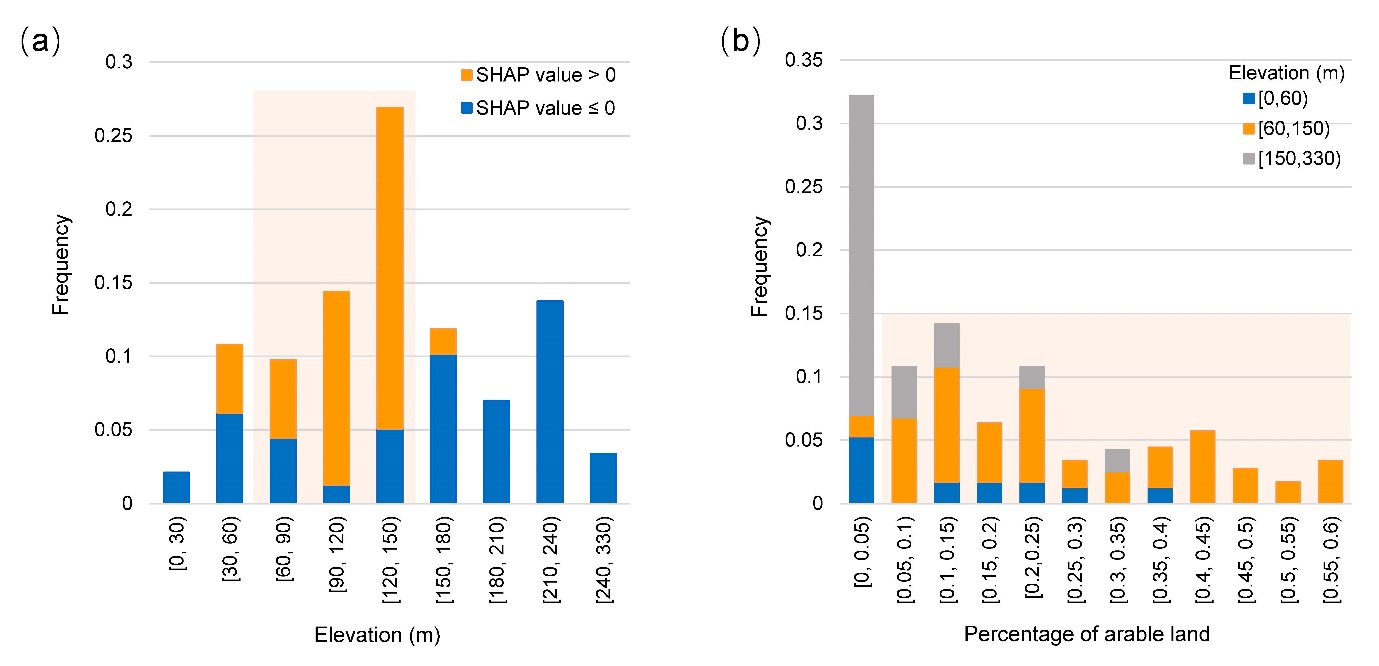


**Fig. S3.** The frequency distribution histograms of (a) the elevation and (b) the percentage of arable land at different elevations.


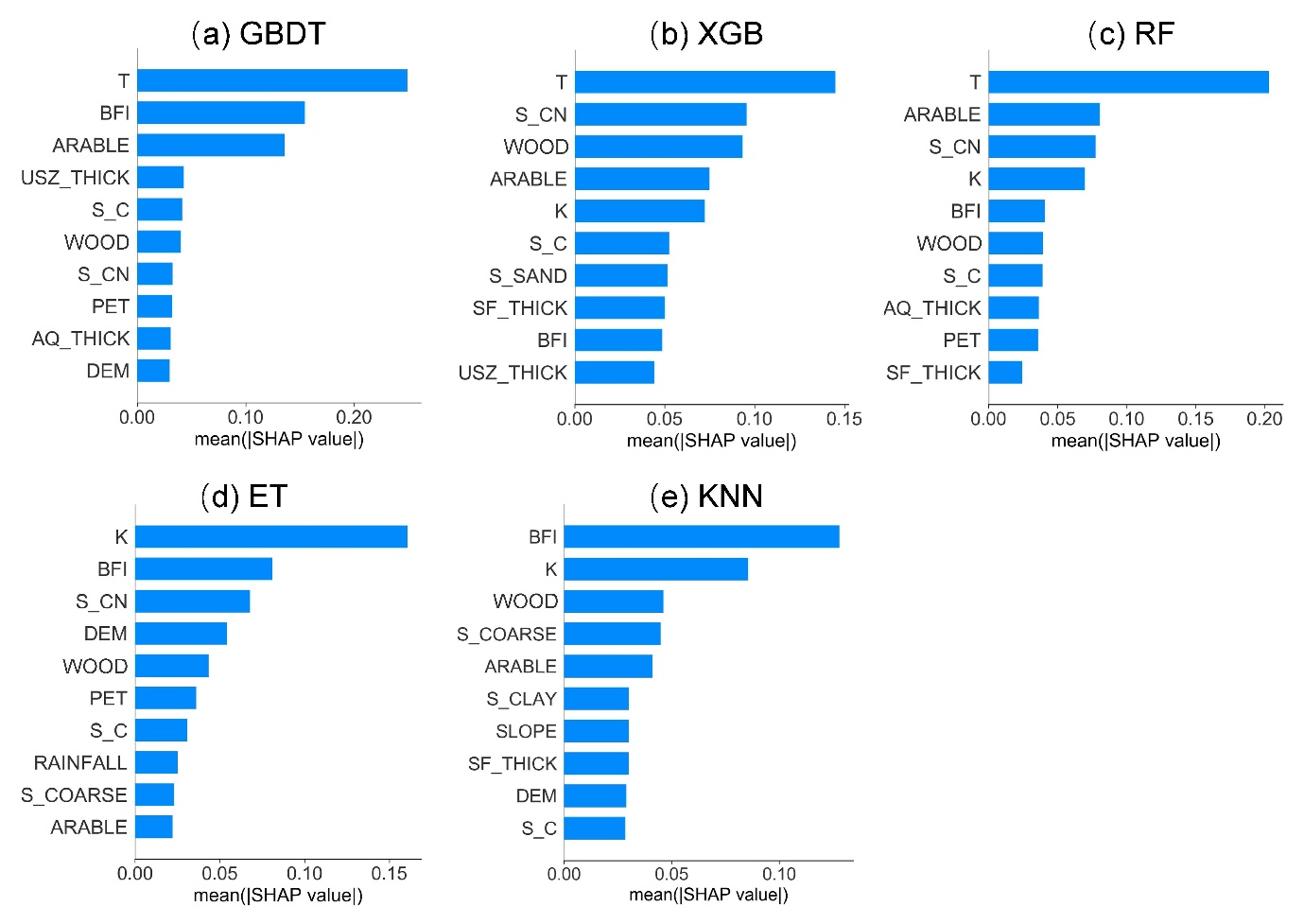


**Fig. S4.** Average absolute value of SHAP values of the top ten most important variables in the base models, including (a) gradient boosting decision tree (GBDT), (b) extreme gradient boosting (XGB), (c) random forest (RF), (d) extremely randomized trees (ET), and (e) k-nearest neighbors (KNN).

**References:**

Ballabio, C., Panagos, P., & Monatanarella, L. (2016). Mapping topsoil physical properties at European scale using the LUCAS database. *Geoderma*, 261, 110-123. https://doi.org/10.1016/j.geoderma.2015.07.006.

BGS. (2020). BGS geology 50k (DigMapGB-50). British Geological Survey. https://www.bgs.ac.uk/datasets/bgs-geology-50k-digmapgb/.

Boorman, D. B., Hollis, J. M., & Lilly, A. (1995). *Hydrology of soil types: a hydrologically based classification of the soils of the United Kingdom* (Report No. 126). Institute of Hydrology. https://nora.nerc.ac.uk/id/eprint/7369/1/IH_126.pdf.

Met Office, Hollis, D., McCarthy, M., Kendon, M., Legg, T., & Simpson, I. (2018). HadUK-Grid gridded and regional average climate observations for the UK. Centre for Environmental Data Analysis. 2023-08-04. http://catalogue.ceda.ac.uk/uuid/4dc8450d889a491ebb20e724debe2dfb.

Morton, R. D., Rowland, C. S., Wood, C. M., Meek, L., Marston, C. G., & Smith, G. M. (2014). Land cover map 2007 (25m raster, GB) v1.2. NERC Environmental Information Data Centre. https://doi.org/10.5285/a1f88807-4826-44bc-994d-a902da5119c2.

Osório, B., Redhead, J. W., Jarvis, S. G., May, L., & Pywell, R. F. (2019). CEH Land Cover plus: Fertilisers 2010-2015 (England). NERC Environmental Information Data Centre. https://doi.org/10.5285/15f415db-e87b-4ab5-a2fb-37a78e7bf051.

Reis, S., Liska, T., Steinle, S., Carnell, E., Leaver, D., Roberts, E., Vieno, M., Beck, R., & Dragosits, U. (2017). UK gridded population 2011 based on census 2011 and land cover map 2015. NERC Environmental Information Data Centre. https://doi.org/10.5285/0995e94d-6d42-40c1-8ed4-5090d82471e1.

Wang, L., & Burke, S. P. (2017). A catchment-scale method to simulating the impact of historical nitrate loading from agricultural land on the nitrate-concentration trends in the sandstone aquifers in the Eden Valley, UK. *Science of The Total Environment*, 579, 133-148. https://doi.org/10.1016/j.scitotenv.2016.10.235.
